# Irradiated Bladder Cancer Cells Expressing both GM-CSF and IL-21 versus Either GM-CSF or IL-21 Alone as Tumor Vaccine in a Mouse Xenograft Model

**DOI:** 10.1155/2019/8262989

**Published:** 2019-08-05

**Authors:** Junming Peng, Liefu Ye, Tao Li, Qingguo Zhu, Jinan Guo, Kefeng Xiao, Yongbao Wei

**Affiliations:** ^1^Department of Urology, The Second Clinical Medical College of Jinan University/Shenzhen People's Hospital, Shenzhen, Guangdong 518000, China; ^2^Department of Urology, Fujian Provincial Hospital, Fuzhou, Fujian 350001, China

## Abstract

Previous studies have established the efficacy of irradiated cancer cells overexpressing GM-CSF or IL-21 as a vaccine. Here we examined whether the vaccine efficacy was greater when both factors were overexpressed together. MB49 bladder cancer cells were transfected with expression plasmid pT7TS encoding mouse GM-CSF and human IL-21, and then irradiated with 100 Gy at 4 days later. The cells (1×10^7^ per animal) were injected subcutaneously into C57BL/6 mice at 0, 4, 8, and 12 days after inoculation with MB49 tumor xenografts. Control animals were injected with MB49 cells transfected with pT7TS encoding GM-CSF or IL-21 on its own. Tumor growth was monitored for 45 days and compared among the groups using repeated-measures ANOVA. Vaccination with irradiated MB49 cells did not affect xenograft growth. Vaccination with irradiated cells overexpressing GM-CSF or IL-21 alone significantly inhibited tumor growth and led to significantly more CD4^+^ CD8^+^ T cells and fewer CD4^+^ Foxp3^+^ T cells in the spleen and xenograft. These effects were even greater following vaccination with irradiated cells overexpressing both GM-CSF and IL-21. Irradiated bladder cancer cells overexpressing both GM-CSF and IL-21 are more effective than cells expressing either factor alone as a vaccine against bladder cancer.

## 1. Introduction

Bladder cancer is one of the most common urologic malignancies. Treatments include surgery and cytotoxic chemotherapy, but long-term disease-free survival remains low [1]. Therefore, novel therapies are needed to address and prevent recurrence of bladder cancer. Immunotherapy has become an increasingly promising possibility in cancer treatment. There is growing evidence that a vaccine-based approach can treat a range of cancers [2, 3].

Bladder cancer is known to trigger immune dysfunction, particularly with lymphocytes [4]_._ Identification of tumor-associated antigens that elicit antitumor responses from T cells is a challenge in developing bladder cancer vaccines. In order to enhance antitumor immunity, cytokines are used to improve tumor antigen presentation or enhance T cell response to cancer cells.

Granulocyte-macrophage colony-stimulating factor (GM-CSF) has been used in many studies as an adjuvant to enhance antitumor immunity. It can enhance the immunogenicity of tumor cells and effectively promote uptake of tumor-associated antigens [5, 6], but it sometimes proves ineffective and tumors continue to grow. This may occur when the GM-CSF fails to generate sufficient tumor-specific T cells, or when these cells are inactive at the tumor site [7]. At the same time, patients with bladder cancer exhibit an impaired T-cell response. Circulating T cells from bladder cancer patients are much less responsive to polyclonal activation than T cells from healthy donors [4]. We hypothesized that we might improve the number and activity of GM-CSF-stimulated T cells by exposing them also to a T cell growth factor.

One such growth factor may be interleukin-21 (IL-21), a member of the *γ*-chain family of cytokines. IL-21 promotes a memory phenotype in activated T cells and suppresses regulatory T cells (Tregs), thereby strengthening the T cell response [8–10]. At the same time, clinical trials suggest that IL-21 is well tolerated by patients and triggers only mild toxicity [11]. These features make IL-21 a potent antitumor agent. However, its activity must be carefully controlled, since the immunostimulatory IL-21 can potently induce apoptosis in conventional dendritic cells via a mechanism dependent on STAT3 and BIM, and it can upregulate the immunosuppressor IL-10 [12, 13].

In the present study, we sought to determine whether the ability of GM-CSF to stimulate antitumor immunity can be potentiated using IL-21. A vaccine of irradiated bladder cancer cells coexpressing GM-CSF and IL-21 was compared with vaccines expressing either factor on its own for the ability to prevent growth of mouse bladder cancer tumors. Changes in tumor microenvironment associated with the different expression conditions were investigated.

## 2. Materials and Methods

### 2.1. Materials

The mouse bladder cancer cell line MB49 was derived from mouse urothelial carcinoma cells. The melanoma cell line was provided by Dr. I. C. Summerhayes at the Lahey Clinic (Burlington, MA, USA). Both cell lines were tested every six months. C57BL/6 mice 8 weeks old were purchased from the Experimental Animal Center at Southern Medical University (Guangzhou, China). MB49 cells were cultured in RPMI 1640 culture medium supplemented with 10% fetal bovine serum. The eukaryotic expression plasmid pT7TS (3 kb) was purchased from Invitrogen (Alpha Innotech, San Leandro, CA, USA). Primers were designed according to the* NDV F* gene sequence and synthesized by GENEWIZ (China). All procedures were carried out in accordance with relevant laws and institutional guidelines and were approved by the institutional Animal Care and Use Committee of Fujian Provincial Hospital(K2017-03-074).

### 2.2. Primer Synthesis and PCR Amplification

Reference sequences for amplifying the GM-CSF and IL-21 coding regions were the cDNA sequences of mouse GM-CSF and human IL-21 in GenBank. The primer sequences for mouse GM-CSF were P1 (sense), 5′-AAGCTTCTGAGGAGGATGTGGCTGC-3'; and P2 (antisense), 5′-GGATCCTTTTTGGCCTGGTTTTTTG-3′. The primer sequences for human IL-21 were P3 (sense), 5′-GGAAGCTTAGGATGGGCAGATCCAGTCCTGGCAAC-3′; and P4 (antisense), 5′-CGGGATCCTCAGGAATCTTCACTTCCGTG-3′. All primers were synthesized by Shanghai Boya Biotechnology (Shanghai, China).

### 2.3. Construction of GM-CSF/IL-21 Expression Plasmids

Using plasmid PT7-GM-CSF as template, primers P1 and P2 were used to amplify the GM-CSF coding sequence (named PCR1) in a 20-*μ*l PCR reaction, and the amplicon was ligated into PT Simple Vector and transformed into top10 competent bacteria. Transformed bacteria were cultured in LB broth containing ampicillin, and plasmid DNA was prepared from single colonies. Positive clones were identified through DNA digestion analysis with one and two restriction enzymes, and the DNA was verified by sequencing. The resulting plasmid was designated PT7-GM-CSF-T.

Similarly, PT7-IL-21 was used as template to amplify the IL-21 coding sequence (referred to as PCR2) using primers P3 and P4. PT-GM-CSF-T was cut with* Bam*HI and* Xho*I, and the larger fragment was ligated with PCR2. The ligation products were transformed into top10 competent bacteria. Clones positive for PT-GM-CSF/IL-21 were identified based on digestion with* Hind*III and* Xho*I. PT-GM-CSF/IL-21 was transformed into* E. coli *DH5*α*, and ampicillin-resistant clones were cultured in LB broth containing ampicillin. Plasmids were prepared using the Plasmid Midi Kit and checked using restriction analysis and sequencing.

### 2.4. Transfection

Cells were cultured in RPMI 1640 containing 10% fetal bovine serum. Before experiments, adherent cells were digested with trypsin, resuspended in serum-containing medium, seeded in 6-well plates (3×10^5^ cells/well), and cultured at 37°C with 5% CO_2_ for 24 h. When cells were 80-90% confluent, MB49 cells were transfected with plasmids (4 *μ*g) using Lipofectamine 2000 according to the manufacturer's instructions (Invitrogen). Transfections were performed in triplicate. Control cells were mock-transfected or transfected with empty plasmid. At 24, 48, 72, and 96 h after transfection, supernatant was collected and GM-CSF/IL-21 levels were determined using kits from Abcam (Cambridge, U.K).

### 2.5. Preparation of Cancer Cell Vaccines

MB49 cells transfected with plasmids expressing GM-CSF, IL-21, or both factors were cultured for four days and then irradiated with 6000 cGy ^60^Co. Cells were resuspended in phosphate-buffered saline (PBS) at a concentration of 1×10^7^/mL for future use.

### 2.6. Western Blotting Analysis of Protein Expression in the Different Vaccines

Conditioned medium from cultured MB49 cells overexpressing mouse GM-CSF and/or human IL-21 was filtered through a 0.45-*μ*m filter. The total protein was subjected to SDS-PAGE, transferred to nitrocellulose membrane and incubated with the corresponding antibodies. After overnight incubation with primary antibodies at 4°C, the membranes were washed with PBST containing 0.05% Tween-20 and incubated with HRP-conjugated secondary antibodies at room temperature for 1 h. Protein bands of interest were detected by enhanced chemiluminescence substrates (Perkin Elmer) and images were captured by FluorChem FC2 (Alpha Innotech, San Leandro, CA, USA).

### 2.7. Xenograft Experiments

Adult C57BL/6 mice 8 weeks old were injected subcutaneously with MB49 cells (1×10^6^) into the left hind leg. On days 0, 4, 8, and 12 after inoculation, mice were randomized to receive subcutaneous injection of PBS or irradiated MB49 cells (1×10^7^) that had been mock-transfected or transfected with plasmid expressing GM-CSF/IL-21, GM-CSF, or IL-21 (n=12 per group). Tumor growth was monitored every 5 days. Tumor volume was calculated using the formula V= 0.5 × L × W2, where L and W are tumor length and width. Tumor wet weight was also measured.

### 2.8. Tumor-Specific Cytotoxicity Assay

At 1 week after the last vaccine dosing, spleens were harvested to measure the cytotoxicity of tumor-specific cytotoxic T lymphocytes (CTLs). Briefly, isolated splenocytes were cocultured 5:1 with MB49 cells treated with mitomycin C (50 mg/mL); the mitomycin C acted as stimulators. Cultures were restimulated on day 2 by adding recombinant human IL-2 (20 U/mL) to the medium. Five days later, dead cells were removed by centrifugation. CTL activity was quantified using the CytoTox 96 Non-Radioactive Cytotoxicity Assay (Promega). The target cells were MB49 cells present at certain effector/target ratios. After CTLs had been cultured with tumor cells for 4-5 h at 37°C, the supernatant was removed and incubated for 30 min at room temperature with substrate in order to assay for release of lactate dehydrogenase. Total cytotoxicity was calculated as follows:

(1) %Cytotoxicity=Experimental−Effector  Spontaneous−Target  SpontaneousTarget  Maximum−Target  Spontaneousα×100

On day 60 after therapeutic vaccination, the specific immune response in surviving GM-CSF/IL-21 mice was assessed. Animals were injected subcutaneously with MB49 cells (1 × 10^6^) in the left hind leg and the same number of melanoma cells in the right hind leg.

### 2.9. Flow Cytometry of Dendritic Cells in the Spleen

One week after the last vaccine dosing, splenocytes were isolated, red blood cells were lysed, and remaining cells were washed in PBS containing 1% bovine serum albumin at room temperature. Washed cells were stained for 30 min at room temperature with PE-labeled anti-mouse CD11c and FITC-labeled anti-mouse CD80 antibodies (Biolegend, San Diego, CA, USA). Double-positive cells considered to be mature dendritic cells were measured by flow cytometry (Becton Dickinson, San Jose, CA, USA).

### 2.10. Flow Cytometry of T Cell Subsets in Blood

To determine the effect of therapeutic vaccines on different T cell subsets in mice, blood (100 mL) was collected at the indicated time points in 12 mice/group. Total number of lymphocytes was determined. CD4^+^ and CD8^+^ T cell populations were determined on days 12 and 27 by staining with PE-labeled anti-mouse CD4 and FITC-labeled anti-mouse CD8 antibodies (eBioscience, San Diego, CA). Then CD4^+^ and CD8^+^ T cell populations were measured by flow cytometry. To determine IFN-r^+^ CD8^+^ and CD4^+^ Foxp3^+^ T cell populations, cells were stained with FITC-labeled anti-mouse CD8 or FITC-labeled anti-mouse CD4 (Clone RM4-5, concentration 0.5 mg/m, Biolegend), fixed, permeabilized, and stained with PE-labeled anti-IFN-*γ* or PE-labeled anti-FoxP3 antibody (Biolegend). T cell subsets were measured using flow cytometry. All experiments were carried out in triplicate.

### 2.11. Histology and Immunohistochemistry

Tissue sections (6-7 mm) were fixed in -20°C cold acetone for 15 min and then incubated for 1 h at 37°C with anti-mouse CD4 antibody (Bioworld, New Brunswick, NJ, USA) or anti-mouse CD8 (Epitomics, Burlingame, CA, USA). Binding of these primary antibodies was visualized using the anti-rat Ig SABC kit (Spring Bioscience, Pleasanton, CA, USA) and counterstained with hematoxylin-eosin. Brown and yellow staining represented positive cells.

### 2.12. Assay of Serum IL-4, IL-12, IL-10, and IFN-*γ*

On day 27 after tumor injection, 100 ml blood was collected from each experiment subject and congealed at room temperature for 20 minutes; then the supernatant was harvested by centrifugation at 3000 rpm for 5 minutes. Concentrations of IL-4, IL-12, IL-10, and IFN-*γ* in serum were determined using an ELISA kit (R & D Systems, Minneapolis, MN, USA).

### 2.13. Statistical Analysis

Continuous data are presented as mean ± standard deviation. Survival among different groups was compared using Kaplan-Meier analyses, followed by the log-rank test. Inter-group differences in tumor growth were assessed for significance using repeated-measures ANOVA, followed by the LSD test for pairwise comparison. All statistical analyses were performed using SPSS 22.0 (IBM, Chicago, IL, USA). P< 0.05 was defined as significant.

## 3. Results and Discussion

### 3.1. Amplification of Target Vaccine Genes and Expression in Transfected Cells

**Figure 1:**
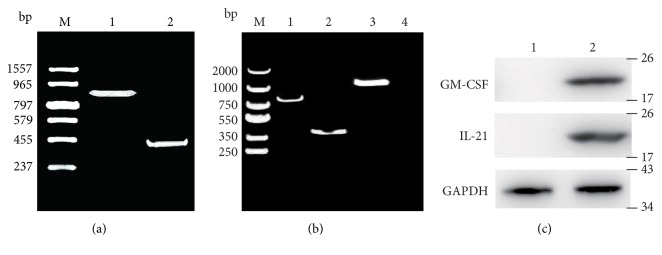
*RT-PCR and Western blotting analysis of the construct plasmid*. (a) PCR amplification of target genes. M, DL2000 DNA marker. Lane 1, GM-CSF. Lane 2, IL-21. (b) RT-PCR analysis of the fusion GM-CSF and IL-21 in cells transfected with indicated plasmid. M, DL2000 DNA marker. Lane 1, GM-CSF. Lane 2, IL-21. Lane 3, GM-CSF/IL-21. (c) Western blotting analysis of MB49 cells transfected with Vector and PT-GM-CSF /IL-21plasmid.

The coding sequences for mouse GM-CSF and human IL-21 were amplified using primer pairs P1/P2 and P3/P4, respectively, yielding amplicons of 0.88 and 0.43 kb (Figure 1(a)). Cells were transfected with PT-GM-CSF/IL-21, and total cellular RNA was extracted from cells and used as template in reverse transcription (RT)-PCR using primers P1 and P2. A specific band of approximately 0.86 kb was detected, consistent with expression of GM-CSF (Figure 1(b)). When the RT-PCR was performed with primers P1 and P4, a band of approximately 1.31 kb was detected, consistent with expression of the fused GM-CSF and IL-21 coding regions. In addition, Western blotting was also performed to confirm GM-CSF and IL-21 expression in MB49 cells transfected as indicted with Lipofectamine 2000 (Figure 1(c)).

### 3.2. GM-CSF/IL-21 Vaccine Inhibits Xenograft Growth

**Figure 2:**
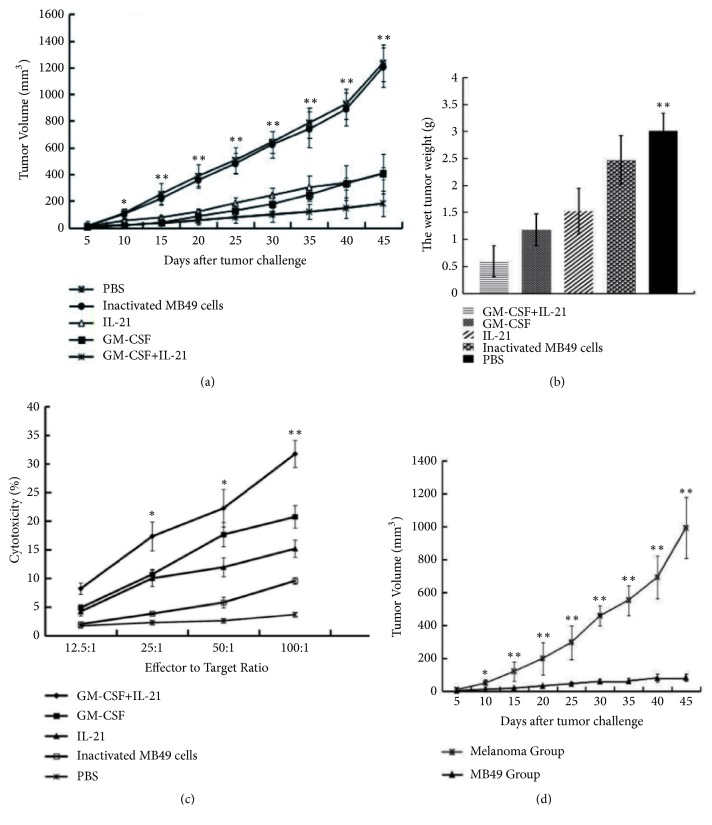
*Antitumor efficacy of GM-CSF/IL-21 vaccine*. (a, b) After establishing a metastatic mouse model of bladder cancer, therapeutic vaccines were injected into the mice at 0, 4, 8, and 12 days after xenograft tumor inoculation (*P* < 0.05). (c, d) Tumor-specific cytotoxicity assay* in vitro *and* in vivo* (*P* < 0.05).

After vaccination, tumor volume and weight in GM-CSF/IL-21 animals differed significantly from the values in other treatment groups (Figures 2(a) and 2(b)). Inactivated MB49 cells did not produce antitumor effects. Xenogrfat growth was significantly slower in mice receiving the GM-CSF/IL-21-expressing vaccine than in mice receiving inactivated cells expressing either GM-CSF or IL-21.

### 3.3. GM-CSF/IL-21 Vaccine Induces Tumor-Specific T cell Immunity

Tumor-specific CTL activity in the GM-CSF/IL-21 group was significantly different from that in the other groups (Figure 2(c)). After day 60, MB49 tumor volume in the left hind leg was significantly smaller than melanoma volume in the right hind leg (P< 0.05; Figure 2(d)). This indicates that the GM-CSF/IL-21 vaccine can effectively protect mice against challenge with MB49 cells but not melanoma cells.

### 3.4. GM-CSF/IL-21 Vaccine Increases the Numbers of Mature Dendritic, CD8^+^, and CD4^+^ Effector T Cells but Decreases Tregs

**Figure 3:**
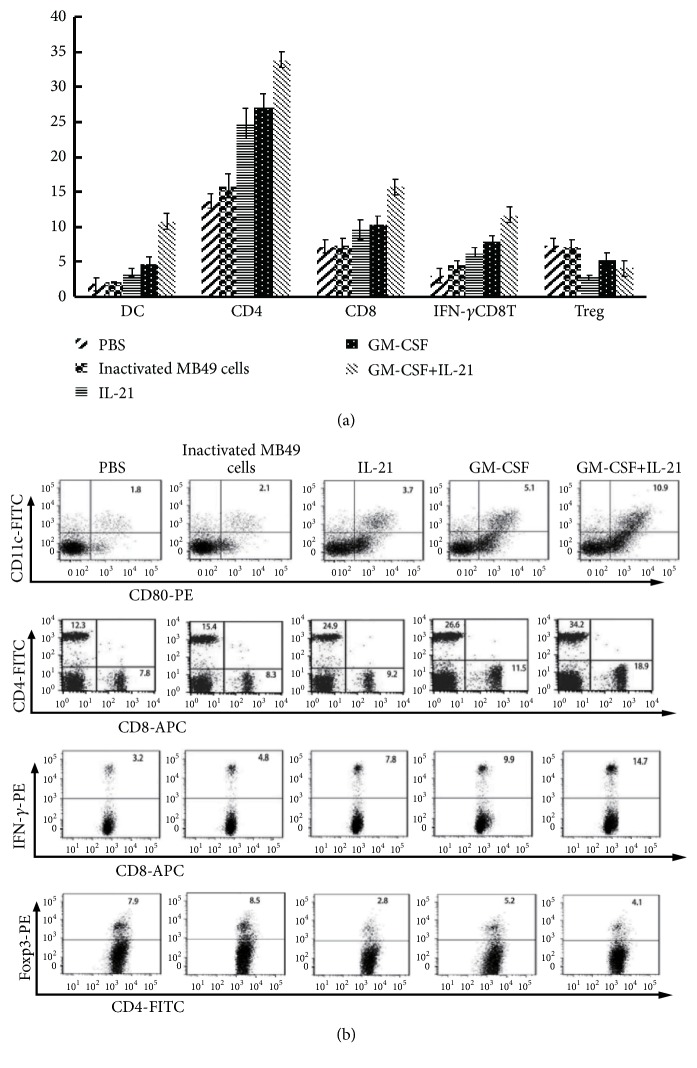
*Flow cytometry of dendritic cells and T cell subsets*. (a) Percentages of dendritic cells and subsets of CD4^+^, CD8^+^, IFN-rCD8^+^, and CD4^+^ Foxp3^+^ T cells in mice on day 19 after vaccination (*P* < 0.05). (b) Typical data from a representative experiment in panel (a) (n = 12).

At one week after the last vaccine dosing, the number of mature (CD11c^+^ CD80^+^) dendritic cells was greater in GM-CSF/IL-21 mice than in other animals (Figures 3(a) and 3(b)). Similarly, the numbers of CD4^+^ and CD8^+^ T cells were higher in the GM-CSF/IL-21 group (P< 0.05; Figures 3(a) and 3(b)). The GM-CSF/IL-21 group also showed the highest proportion of IFN-rCD8^+^ T cells and the lowest proportion of CD4^+^ Foxp3^+^ T cells.

### 3.5. GM-CSF/IL-21 Vaccine Increases Tumor-Infiltrating CD8^+^ and CD4^+^ Effector T Cells

**Figure 4:**
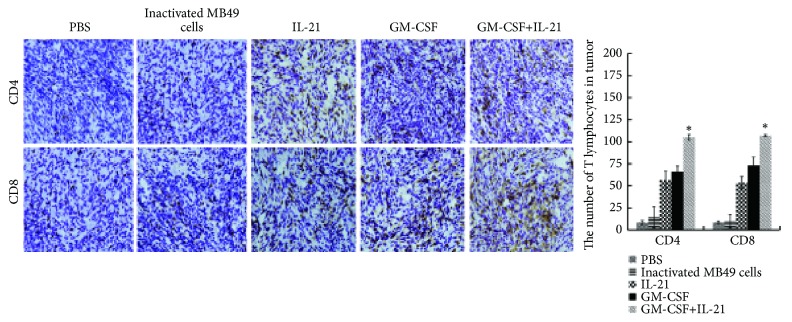
*GM-CSF/IL-21 enhanced the infiltration by CD4* ^*+*^ *and CD8* ^*+*^ *T cells and upregulated IL-12 and IFN-γ (P < 0.05)*. On day 19 after tumor injection, subcutaneous tumor tissue was collected from each group, and CD4^+^ and CD8^+^ T cells were detected by histology and immunohistochemistry (*P* < 0.05). Magnification, 200×.

The GM-CSF/IL-21 group showed more CD4^+^ and CD8^+^ T cells that had infiltrated into the tumor site than the other groups (P< 0.05; Figure 4(a)).

### 3.6. GM-CSF/IL-21 Vaccine Upregulates IL-12 and IFN-*γ* While Keeping IL-10 Relatively Low

**Figure 5:**
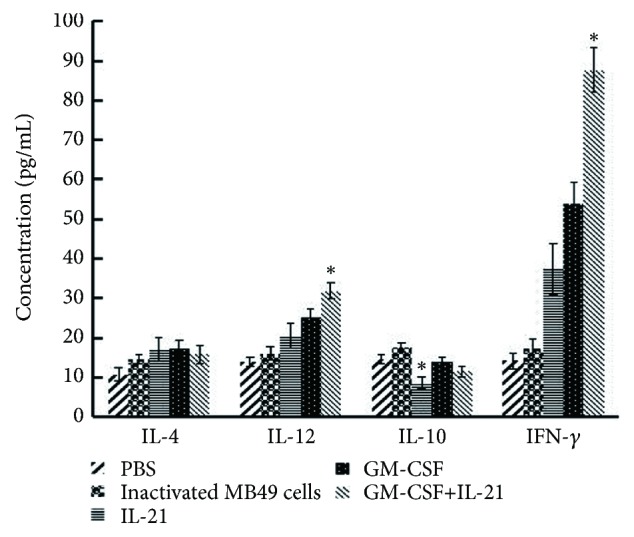
*Serum concentration of IL-4, IL-12, IL-10, and IFN-γ*. On day 19 after tumor injection, serum levels were measured using ELISA (*P* < 0.05).

IL-10 is a key immunosuppressor [14], while IL-12 and IFN-*γ* inhibit tumor cell growth [15, 16]. IL-21 can elevate IL-10 expression [13], and indeed the IL-10 concentration was highest in the IL-21 group (P< 0.05; Figure 5). The GM-CSF/IL-21 group showed the highest concentrations of IL-12 and IFN-*γ* but relatively low IL-10 concentration (P< 0.05; Figure 5).

## 4. Discussion

In the present study, we have shown that vaccination with tumor cells coexpressing GM-CSF and IL-21 elicited potent antitumor responses in a mouse model of bladder cancer. This vaccine inhibited tumor growth and effectively induced specific antitumor immunity* in vitro* and* in vivo.* Vaccination with tumor cells expressing either cytokine on its own showed much lower efficacy. These studies indicate that GM-CSF and IL-21 can interact synergistically to protect mice from bladder cancer, and that this cell-based vaccination approach is safe in mice.

Whole-cell vaccination, a promising strategy in immunotherapy, provides multiple identified and unidentified tumor-associated antigens (TAAs) that activate CD4^+^ and CD8^+^ CTLs, thereby minimizing immune escape [17]. This strategy is thwarted by the ability of tumors to downregulate TAA expression, highlighting the need to identify new TAAs for developing tumor vaccines. In addition to triggering weak immune responses, tumor cells may actually suppress immune responses by influencing signaling mediated by transforming growth factor-*β*, which induces Tregs differentiation and inhibits dendritic cell maturation [18]. Overcoming immune evasion requires the use of adequate immune costimulatory agents as adjuvants.

Novel combination therapies in cancer immunotherapy treatment can lead to greater antitumor effects or fewer side effects than monotherapies [19, 20]. The synergy between GM-CSF and IL-21 may be due in part to the ability of GM-CSF to stimulate dendritic cell maturation, which enhances the immunogenicity of tumor cells and effectively promotes TAA uptake [5]. After dendritic cell maturation and onset of T cell immune response, IL-21 promotes and maintains the activity of tumor-specific T cells [10]. Our study showed that GM-CSF/IL-21 vaccine elicited antitumor effects to a greater extent than the vaccines expressing either cytokine on its own. These stronger effects positively correlated with enhanced infiltration by CD4^+^ and CD8^+^ effector T cells: numbers of CD4^+^, CD8^+^, and IFN-*γ*^+^ CD8^+^ T cells in blood and tumor were higher in the GM-CSF/IL-21 group than in the other groups. The presence of CD8^+^ T cells can predict survival in muscle-invasive bladder cancer [4], and high intratumoral CD8^+^ T cell density is a significant predictor of favorable overall survival [21].

In our study, mice treated with the GM-CSF/IL-21 vaccine contained a greater number of mature dendritic cells in the spleen than animals treated with the IL-21 vaccine. Fewer CD4^+^ Foxp3^+^ T cells were detected in the GM-CSF/IL-21 group than in the GM-CSF group. Our results indicate that GM-CSF and IL-21 synergistically interact to enhance antitumor effects, and an extensive literature supports this idea. GM-CSF has been shown to generate myeloid suppressor cells in humans, causing activation of CD4^+^ CD25^+^ Tregs [22]. Greater numbers of Tregs can reduce the numbers of mature dendritic cells and affect the interaction between dendritic and T cells either directly by modulating their contact with each other or indirectly by changing the immune microenvironment [23]. IL-21 can enhance immune responses by suppressing Tregs (CD4^+^ Foxp3^+^) [11], yet it can also immunosuppress by inducing apoptosis of conventional dendritic cells [12]. GM-CSF blocks IL-21-mediated apoptosis of dendritic cells by activating primarily STAT5 instead of STAT3 and by inhibiting BIM induction [12]. These mechanistic studies likely help explain why GM-CSF and IL-21 interacted synergistically in our study. Indeed, fusing GM-CSF and IL-21 into a single bifunctional cytokine showed synergistic anticancer immune effects, because each cytokine promotes the other's binding to its receptor complexes [24]. To our knowledge, the present study is the first report showing that combining GM-CSF with IL-21 enhances vaccine efficacy against bladder cancer.

Some cytokines may mediate the synergy between GM-CSF and IL-21. Our results revealed that the serum in GM-CSF/IL-21 animals contained the highest concentrations of IFN-*γ* and IL-12 but relatively low IL-10. IFN-*γ* and IL-12 play an important role in the antitumor immune response [15, 16], while IL-10 is an immunosuppressive factor involved in tumorigenesis [14]. Further studies should examine the factors and signaling steps that help mediate antitumor synergy between GM-CSF and IL-21.

## 5. Conclusions

In summary, our results suggest that coexpression of GM-CSF and IL-21 in a whole-cell cancer vaccine can enhance efficacy against bladder cancer in mice. The vaccine stimulated CD4^+^ and CD8^+^ T cell-dependent adaptive responses and generated specific antitumor immunity. These findings may help guide development of effective vaccines against bladder cancer.

## Data Availability

The data used to support the findings of this study are available from the corresponding author upon request.
